# Prevalence of hypertriglyceridemia among Royal Thai Army personnel and its related cardiometabolic risk factors, from 2017 to 2021

**DOI:** 10.1186/s12889-022-13992-2

**Published:** 2022-08-17

**Authors:** Boonsub Sakboonyarat, Jaturon Poovieng, Kanlaya Jongcherdchootrakul, Phutsapong Srisawat, Panadda Hatthachote, Mathirut Mungthin, Ram Rangsin

**Affiliations:** 1 Department of Military and Community Medicine, Phramongkutklao College of Medicine, Bangkok, 10400 Thailand; 2 Department of Medicine, Phramongkutkalo College of Medicine, Bangkok, 10400 Thailand; 3 Department of Physiology, Phramongkutklao College of Medicine, Bangkok, 10400 Thailand; 4 Department of Parasitology, Phramongkutklao College of Medicine, Bangkok, 10400 Thailand

**Keywords:** Hypertriglyceridemia, Behavioral risk, Cardiometabolic risk, RTA, Thailand

## Abstract

**Background:**

Hypertriglyceridemia is a common health problem independently associated with an increased risk of atherosclerosis cardiovascular diseases (ASCVD), including ischemic heart disease and stroke. This study aims to determine the prevalence of hypertriglyceridemia among Royal Thai Army (RTA) personnel and its behavioral and cardiometabolic risk factors using the RTA personnel database of the physical health examination from 2017 to 2021.

**Methods:**

A serial cross-sectional study was conducted from 2017 to 2021. A total of 257,683 active-duty RTA personnel aged 35–60 years were included in the study. We defined hypertriglyceridemia as fasting triglyceride ≥150 mg/dL. Moreover, we performed a multivariable logistic regression analysis to investigate behavioral and cardiometabolic risk factors for the prevalence of hypertriglyceridemia. The magnitude of the association was presented as an adjusted odds ratio (AOR) with a 95% confidence interval (CI).

**Results:**

The hypertriglyceridemia prevalence among RTA personnel was 43.4% (95% CI: 42.9–43.8%) in 2017. It then continuously decreased to 40.3% (95% CI: 39.9–40.7%) in 2020 and slightly rose to 41.0% (95% CI: 40.6–41.4%) in 2021 (*p* for trend < 0.001). The prevalence of hypertriglyceridemia was higher for males than females (AOR 2.15; 95% CI: 2.07–2.23); RTA personnel aged 40–44 years compared with those aged 35–39 years (AOR 1.05; 95% CI: 1.02–1.08); and RTA personnel residing in the northeast (AOR; 1.15 95% CI: 1.11–1.18) and the north (AOR 1.05; 95% CI: 1.02–1.08) compared with those residing in Bangkok. The independent behavioral factors associated with hypertriglyceridemia included alcohol consumption, smoking, and sedentary behavior. Moreover, cardiometabolic risk factors, including higher body mass index, high fasting plasma glucose (≥ 100 mg/dL), high blood pressure (≥ 140/90 mmHg), and hypercholesterolemia (≥ 200 mg/dL), were significantly related to hypertriglyceridemia.

**Conclusion:**

Our data demonstrated that hypertriglyceridemia is a frequent health issue, especially among males, participants aged 40–44 years, and RTA personnel residing in the northeast and the north. The prevalence of hypertriglyceridemia in this population was greatly influenced by alcohol consumption, cigarette smoking, and sedentary behavior. Both behavioral and cardiometabolic risk factors are potential targets for intervention to enhance the primary prevention of sequelae of hypertriglyceridemia, including ASCVD.

## Background

In 2016, the World Health Organization reported that noncommunicable diseases (NCDs) account for 71% of the 57 million global deaths and 31% of those have cardiovascular diseases [1]. Similarly, in Thailand, NCDs are estimated to account for 74% of all deaths, with cardiovascular diseases accounting for one-fourth of them [1]. Hyperlipidemia is a well-documented risk factor for cardiovascular diseases [2, 3]. Hypertriglyceridemia is a common health problem independently associated with an increased risk of atherosclerosis cardiovascular diseases (ASCVD), including ischemic heart disease and stroke [3, 4]. Furthermore, elevated triglyceride (TG) level is an incremental risk for all-cause mortality among patients with coronary heart diseases [5].

The National Health Survey in China [6], the US [7] and Russia [8] reported the prevalence of hypertriglyceridemia (fasting plasma TG ≥ 150 mg/dL) [9] among adults aged 20 years, accounting for 13.1, 25.9, and 29.2%, respectively. In comparison, the National Health Examination Survey (NHES) in Thailand indicated that the prevalence of hypertriglyceridemia among Thai adults aged ≥ 15 years increased from 31.0% in 2014 (NHES V) [10] to 36.0% in 2019 (NHES VI) [11].

Nationwide, approximately 50,000 Royal Thai Army (RTA) personnel aged at least 35 years participate in yearly health examinations provided by the RTA Medical Department (RTAMED). However, there is little information available about hypertriglyceridemia in RTA personnel, who lead very different lives than Thai civilians. As a result, the researchers set out to identify hypertriglyceridemia among RTA personnel using the RTA personnel database of physical health examinations from 2017 to 2021. Furthermore, we explored the association between behavioral and cardiometabolic risk factors and the prevalence of hypertriglyceridemia among RTA personnel.

## Methods

### Study design and subjects

A serial cross-sectional study was conducted from 2017 to 2021. The dataset was retrieved from the annual health examination database of RTA personnel after obtaining permission from the RTAMED in Bangkok, Thailand [12]. The RTAMED provides yearly health examinations for RTA personnel through the Army Institute of Pathology, Armed Forces Research Institute of Medical Sciences, and Phramongkutklao Hospital located in the Bangkok Metropolitan Area and 36 RTA hospitals nationwide, including 10, 10, 10, and 6 RTA hospitals in central, northern, northeastern, and southern Thailand, respectively. The data of health examinations were reported to the RTAMED in Bangkok. Active-duty RTA personnel between the ages of 35 and 60 were eligible for this study. The RTA personnel with comorbidities such as NCDs were also included. Because we used the collected data, the data for RTA personnel who did not participate in each annual health examination were excluded from the data for that year. In this study, we intended to determine the prevalence of hypertriglyceridemia; thus, the RTA personnel with no TG level record in the data were excluded. In the present study, 257,683 RTA personnel from 2017 to 2021 were eligible for the study.

### Data collection

Annually, the Army Institute of Pathology, Armed Forces Research Institute of Medical Sciences, and 37 RTA hospitals provide health examinations for RTA personnel. A self-report guide was conducted using a standardized case report form to obtain demographic characteristics and behavioral risk factors, including age, sex, smoking status, alcohol consumption, regular exercise, and health schemes. For RTA causal workers, health examinations are also provided; thus, the reported health scheme would have included civil servant medical benefits, social security, and universal coverage.

The annual health examination dataset also included anthropometric measurements of weight, height, systolic blood pressure (SBP), and diastolic blood pressure (DBP). Blood pressure (BP) was measured by a trained operator using an automatic blood pressure monitor in the standardized technique following the Thai guidelines on treating HT [13]. Laboratory data included fasting plasma glucose (FPG), fasting TG, and fasting total cholesterol (TC). We defined hypertriglyceridemia as fasting TG ≥150 mg/dL [9] and hypercholesterolemia defined as TC ≥200 mg/dL [14]. Cardiometabolic risk factors included high FPG, high blood pressure (BP), and high body mass index (BMI). FPG was categorized in three groups: < 100 mg/dL, 100 to 125 mg/dL (impaired FPG), and ≥ 126 mg/dL (hyperglycemia) [15]. High BP was defined as SBP ≥140 mmHg or DBP ≥90 mmHg [16]. BMI was calculated as body weight in kilograms divided by height in meters squared (kg)/(m)^2^. BMI was classified into five groups: < 18.50 kg/m^2^ (underweight), 18.50 to 22.99 kg/m^2^ (normal), 23.00 to 24.99 kg/m^2^ (overweight), 25.00 to 29.99 kg/m^2^ (obese I), and ≥ 30.00 kg/m^2^ (obese II) [17].

### Statistical analysis

We calculated the frequency distribution of demographic, behavioral, and cardiometabolic risk factors to describe the study subjects. Continuous variables, including age, BMI, SBP, DBP, FPG, TG, and TC, were presented as mean and standard deviation (SD). Categorical data were presented as percentages, including sex, age group, region, health scheme, smoking status, alcohol consumption, regular exercise, BMI categories, FPG categories, high BP, and high TC. We calculated the prevalence of hypertriglyceridemia and presented it as a percentage with a 95% confidence interval (CI). *P* for trend was calculated using logistic regression to test the statistical significance of trends in the prevalence of hypertriglyceridemia. Linear regression analysis was used to determine the association between the mean TG and BMI, FPG, SBP, and DBP categories.

Univariable analysis was performed to determine the association between demographic, behavioral, and cardiometabolic risk factors and the prevalence of hypertriglyceridemia. Finally, a multivariable logistic regression model was used to determine independent factors associated with hypertriglyceridemia. The magnitude of the association was presented as an adjusted odds ratio (AOR) with 95% CI. The variables including sex, age, region, health scheme, smoking status, alcohol consumption, regular exercise, BMI, FPG, high BP, TC, and year were included in the final model. A two-sided *p*-value less than 0.05 was considered statistically significant. All statistical analyses were performed using StataCorp. 2021, *Stata Statistical Software: Release 17*, College Station, TX: StataCorp LLC.

### Ethics considerations

The study was reviewed and approved by the Institutional Review Board, Royal Thai Army Medical Department, in compliance with international guidelines such as the Declaration of Helsinki, the Belmont Report, CIOMS Guidelines, and the International Conference on Harmonization of Technical Requirements for Registration of Pharmaceuticals for Human Use-Good Clinical Practice (ICH-GCP) (approval number S067h/64). A waiver of documentation of informed consent was granted because we used secondary data. The Institutional Review Board of the Royal Thai Army Medical Department approved the informed consent waiver.

## Results

### Characteristics of participants

Table 1 shows the demographic, behavioral, and cardiometabolic characteristics of the 257,683 RTA personnel from 2017 to 2021. In all, approximately 90% of participants were males. The mean age of study participants ranged from 46.7 to 47.9 years. One-third of participants resided in central regions. The prevalence of current regular smokers continuously increased from 13.9% in 2017 to 17.4% in 2021. Approximately two-thirds of the study participants reported alcohol consumption over a demi-decade. Regular exercise was reported by 59.1% of the study participants in 2017 and dropped to 53.0% in 2021. The prevalence of the study participants with BMI ≥ 30 kg/m^2^ rose from 9.0 to 10.9% over half a decade. One-fourth of the study participants presented FPG 100–125 mg/dL, while approximately 10% of the study participants presented FPG ≥ 126 mg/dL. Overall, one-fourth of the study participants had high BP. The average TG in the study participants was 170.8 ± 133.2 mg/dL in 2017 and dropped to 163.8 ± 129.6 mg/dL in 2020 and then rose to 166.9 ± 134.3 mg/dL in 2021. The average TC was 215.1 ± 44.3 in 2017, decreased to 209.5 ± 43.9 mg/dL in 2020, and then increased to 213.8 ± 45.5 in 2021.

**Table 1:** Demographic, behavioral, and cardiometabolic characteristics of participants (2017–2021)

Year	2017	2018	2019	2020	2021
Characteristics	n (%)	n (%)	n (%)	n (%)	n (%)
**No. of participants**	42,777	48,846	57,038	58,152	50,870
**Sex**					
Female	3900 (9.1)	5346 (10.9)	5827 (10.2)	6686 (11.5)	4830 (9.5)
Male	38,877 (90.9)	43,500 (89.1)	51,211 (89.8)	51,466 (88.5)	46,040 (90.5)
**Age (years)**					
mean ± SD	47.9 ± 7.1	47.5 ± 7.3	47.4 ± 7.5	47.4 ± 7.7	46.7 ± 7.7
35–39	7281 (17.0)	9151 (18.7)	11,450 (20.1)	12,502 (21.5)	12,837 (25.2)
40–44	8010 (18.7)	9845 (20.2)	11,280 (19.8)	10,568 (18.2)	9303 (18.3)
45–49	6787 (15.9)	7764 (15.9)	8859 (15.5)	9575 (16.5)	8865 (17.4)
50–54	10,662 (24.9)	10,798 (22.1)	11,790 (20.7)	10,601 (18.2)	8385 (16.5)
≥ 55	10,037 (23.5)	11,288 (23.1)	13,659 (23.9)	14,906 (25.6)	11,480 (22.6)
**Regions**					
Bangkok	7325 (17.1)	9960 (20.4)	10,919 (19.1)	11,143 (19.2)	5552 (10.9)
Central	15,336 (35.9)	17,440 (35.7)	19,604 (34.4)	21,405 (36.8)	19,983 (39.3)
Northeast	7249 (16.9)	8440 (17.3)	9431 (16.5)	11,092 (19.1)	8787 (17.3)
North	9917 (23.2)	7429 (15.2)	11,846 (20.8)	9134 (15.7)	11,031 (21.7)
South	2950 (6.9)	5577 (11.4)	5238 (9.2)	5378 (9.2)	5517 (10.8)
**Health scheme**					
Civil servant medical benefits	41,735 (97.6)	47,836 (97.9)	55,987 (98.2)	56,600 (97.3)	50,010 (98.3)
Social Security	695 (1.6)	550 (1.1)	622 (1.1)	1138 (2.0)	751 (1.5)
Universal Coverage	347 (0.8)	460 (0.9)	429 (0.8)	414 (0.7)	109 (0.2)
**Smoking status**					
Never	22,623 (53.5)	26,282 (54.4)	27,573 (49.5)	26,779 (48.1)	26,339 (51.9)
Ex-smoker	9078 (21.5)	8797 (18.2)	13,121 (23.6)	12,823 (23.0)	10,266 (20.2)
Current smoker (irregular)	4668 (11.0)	5319 (11.0)	6535 (11.7)	6318 (11.3)	5335 (10.5)
Current smoker (regular)	5884 (13.9)	7872 (16.3)	8486 (15.2)	9760 (17.5)	8842 (17.4)
**Alcohol consumption**					
Never	10,432 (24.9)	12,319 (25.7)	12,905 (22.7)	11,386 (20.5)	12,953 (25.5)
Ex-drinker	4535 (10.8)	5408 (11.3)	7515 (13.2)	7163 (12.9)	6671 (13.1)
Current drinker (irregular)	24,536 (58.5)	26,810 (55.9)	31,537 (55.5)	31,172 (56.0)	26,380 (52.0)
Current drinker (regular)	2441 (5.8)	3439 (7.2)	4866 (8.6)	5954 (10.7)	4753 (9.4)
**Regular exercise**					
No	17,106 (40.9)	19,195 (40.9)	19,576 (35.1)	24,047 (42.6)	23,812 (47.0)
Yes	24,667 (59.1)	27,790 (59.1)	36,121 (64.9)	32,359 (57.4)	26,882 (53.0)
**Body mass index (kg/m**^**2**^**)**					
mean ± SD	25.1 ± 3.6	25.2 ± 3.7	25.3 ± 3.7	25.3 ± 3.7	25.4 ± 3.8
18.50–22.99	11,211 (26.2)	12,544 (25.7)	14,417 (25.3)	14,921 (25.7)	12,845 (25.3)
< 18.50	687 (1.6)	688 (1.4)	765 (1.3)	782 (1.3)	664 (1.3)
23.00–24.99	10,795 (25.2)	12,298 (25.2)	14,464 (25.4)	14,530 (25.0)	12,750 (25.1)
25.00–29.99	16,217 (37.9)	18,645 (38.2)	21,685 (38.0)	21,879 (37.6)	19,048 (37.4)
≥ 30.00	3867 (9.0)	4671 (9.6)	5707 (10.0)	6040 (10.4)	5563 (10.9)
**Systolic blood pressure (mmHg)**					
mean ± SD	130.5 ± 16.9	130.6 ± 16.9	130.8 ± 16.8	131.1 ± 16.7	131.8 ± 17.1
< 140	31,829 (74.4)	36,399 (74.5)	42,884 (75.2)	43,413 (74.7)	37,208 (73.1)
≥ 140	10,948 (25.6)	12,447 (25.5)	14,154 (24.8)	14,739 (25.3)	13,662 (26.9)
**Diastolic blood pressure (mmHg)**					
mean ± SD	81.4 ± 11.6	81.3 ± 11.7	81.1 ± 11.6	80.9 ± 11.6	81.3 ± 11.8
< 90	32,575 (76.2)	37,803 (77.4)	44,798 (78.5)	46,018 (79.1)	39,559 (77.8)
≥ 90	10,202 (23.8)	11,043 (22.6)	12,240 (21.5)	12,134 (20.9)	11,311 (22.2)
**Fasting plasma glucose (mg/dL)**					
mean ± SD	103.4 ± 36.0	104.0 ± 36.8	105.1 ± 39.5	103.0 ± 33.9	102.5 ± 32.7
< 100	26,033 (65.2)	29,451 (64.5)	32,857 (64.2)	34,928 (65.9)	30,522 (66.7)
100–125	9844 (24.7)	11,580 (25.4)	12,607 (24.6)	12,857 (24.3)	10,929 (23.9)
≥ 126	4022 (10.1)	4595 (10.1)	5744 (11.2)	5219 (9.8)	4303 (9.4)
**Fasting triglyceride (mg/dL)**					
mean ± SD	170.8 ± 133.2	171.4 ± 136.2	168.8 ± 135.3	163.8 ± 129.6	166.9 ± 134.3
< 150	24,222 (56.6)	27,913 (57.1)	33,195 (58.2)	34,743 (59.7)	30,014 (59.0)
≥ 150	18,555 (43.4)	20,933 (42.9)	23,843 (41.8)	23,409 (40.3)	20,856 (41.0)
**Total cholesterol (mg/dL)**					
mean ± SD	215.1 ± 44.3	213.9 ± 44.8	212.1 ± 44.0	209.5 ± 43.9	213.8 ± 45.5
< 200	15,997 (37.4)	18,935 (38.8)	22,865 (40.1)	24,844 (42.7)	19,829 (39.0)
≥ 200	26,780 (62.6)	29,911 (61.2)	34,173 (59.9)	33,308 (57.3)	31,041 (61.0)

### Prevalence of hypertriglyceridemia

Table 2 presents prevalence of hypertriglyceridemia among RTA personnel from 2017 to 2021. The hypertriglyceridemia prevalence among RTA personnel was 43.4% (95% CI: 42.9–43.8%) in 2017, then continuously decreased to 40.3% (95% CI: 39.9–40.7%) in 2020, and slightly rose to 41.0% (95% CI: 40.6–41.4%) in 2021 (*p* for trend < 0.001).

**Table 2:** Prevalence of hypertriglyceridemia among RTA personnel by demographic characteristics from 2017 to 2021

Characteristics	Overall (***n*** = 257,683)		2017 (***n*** = 42,777)		2018 (***n*** = 48,846)		2019 (***n*** = 58,038)		2020 (***n*** = 58,152)		2021 (***n*** = 50,870)		***p***-for trend
	n	% (95% CI)	n	% (95% CI)	n	% (95% CI)	n	% (95% CI)	n	% (95% CI)	n	% (95% CI)	
**Total**	107,596	41.8 (41.6–41.9)	18,555	43.4 (42.9–43.8)	20,933	42.9 (42.4–43.3)	23,843	41.8 (41.4–42.2)	23,409	40.3 (39.9–40.7)	20,856	41.0 (40.6–41.4)	< 0.001
**Sex**													
Female	5295	19.9 (19.4–20.4)	805	20.6 (19.4–21.9)	1096	20.5 (19.4–21.6)	1189	20.4 (19.4–21.5)	1284	19.2 (18.3–20.2)	921	19.1 (18.0–20.2)	0.012
Male	102,301	44.3 (44.1–44.5)	17,750	45.7 (45.2–46.2)	19,837	45.6 (45.1–46.1)	22,654	44.2 (43.8–44.7)	22,125	43.0 (42.6–43.4)	19,935	43.3 (42.8–43.8)	< 0.001
**Age (years)**													
35–39	22,470	42.2 (41.8–42.6)	3163	43.4 (42.3–44.6)	3965	43.3 (42.3–44.3)	4856	42.4 (41.5–43.3)	5126	41.0 (40.1–41.9)	5360	41.8 (40.9–42.6)	< 0.001
40–44	22,083	45.1 (44.6–45.5)	3690	46.1 (45.0–47.2)	4528	46.0 (45.0–47.0)	5151	45.7 (44.7–46.6)	4635	43.9 (42.9–44.8)	4079	43.8 (42.8–44.9)	< 0.001
45–49	18,243	43.6 (43.1–44.1)	3005	44.3 (43.1–45.5)	3420	44.0 (42.9–45.2)	3858	43.5 (42.5–44.6)	4095	42.8 (41.8–43.8)	3865	43.6 (42.6–44.6)	0.139
50–54	21,354	40.9 (40.5–41.3)	4548	42.7 (41.7–43.6)	4495	41.6 (40.7–42.6)	4717	40.0 (39.1–40.9)	4181	39.4 (38.5–40.4)	3413	40.7 (39.7–41.8)	< 0.001
≥ 55	23,446	38.2 (37.8–38.6)	4149	41.3 (40.4–42.3)	4525	40.1 (39.2–41.0)	5261	38.5 (37.7–39.3)	5372	36.0 (35.3–36.8)	4139	36.1 (35.2–36.9)	< 0.001
**Regions**													
Bangkok	16,978	37.8 (37.4–38.3)	2652	36.2 (35.1–37.3)	3874	38.9 (37.9–39.9)	4296	39.3 (38.4–40.3)	4152	37.3 (36.4–38.2)	2004	36.1 (34.8–37.4)	0.340
Central	39,401	42.0 (41.7–42.3)	6994	45.6 (44.8–46.4)	7569	43.4 (42.7–44.1)	8291	42.3 (41.6–43.0)	8356	39.0 (38.4–39.7)	8191	41.0 (40.3–41.7)	< 0.001
Northeast	19,863	44.1 (43.7–44.6)	3323	45.8 (44.7–47.0)	3810	45.1 (44.1–46.2)	4150	44.0 (43.0–45.0)	4722	42.6 (41.7–43.5)	3858	43.9 (42.9–44.9)	< 0.001
North	21,620	43.8 (43.4–44.2)	4332	43.7 (42.7–44.7)	3354	45.1 (44.0–46.3)	5210	44.0 (43.1–44.9)	3999	43.8 (42.8–44.8)	4725	42.8 (41.9–43.8)	0.063
South	9734	39.5 (38.9–40.1)	1254	42.5 (40.7–44.3)	2326	41.7 (40.4–43.0)	1896	36.2 (34.9–37.5)	2180	40.5 (39.2–41.9)	2078	37.7 (36.4–39.0)	< 0.001

**Fig. 1:**
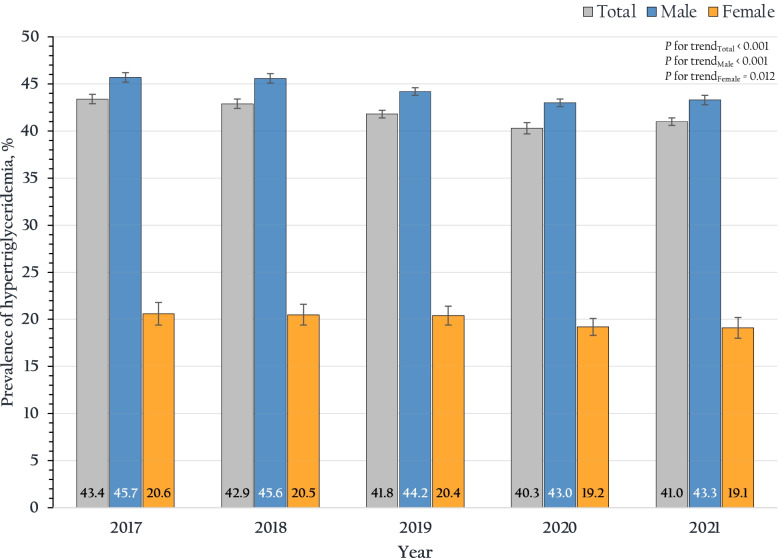
Trends in the prevalence of hypertriglyceridemia and 95% CI from 2017 to 2021

**Fig. 2:**
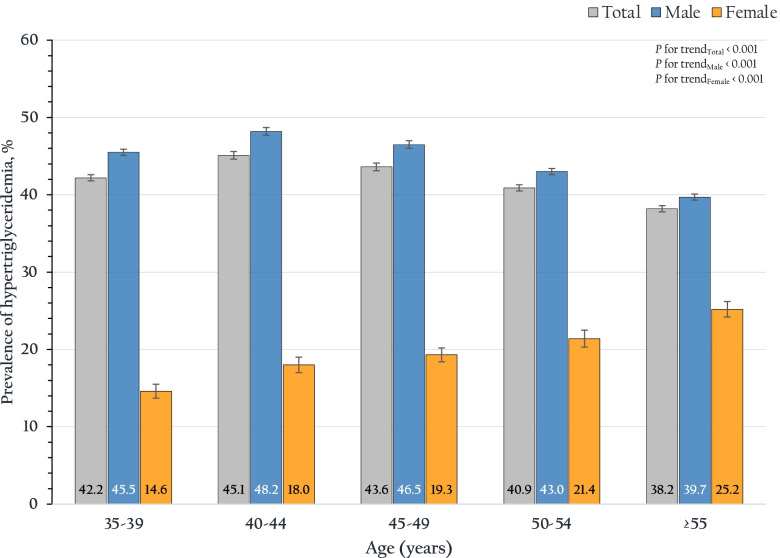
Prevalence of hypertriglyceridemia and 95% CI, stratified by age groups and sex

Figure 1 shows the trends in the prevalence of hypertriglyceridemia among RTA personnel from 2017 to 2021. The overall prevalence of hypertriglyceridemia among males and females was 44.3 (95% CI: 44.1–44.5%) and 19.9 (95% CI: 19.4–20.4%), respectively (*p*-value < 0.001). Figure 2 demonstrates the prevalence of hypertriglyceridemia stratified by age group and sex. Hypertriglyceridemia prevalence among RTA personnel aged 35 to 39 years was 42.2% (95% CI: 41.8–42.6%), was incrementally reaching 45.1% (95% CI: 44.6–45.5%) for those aged 40–44 years, and then continuously declined with the higher age group aged ≥ 55 years accounting for 38.2% (95% CI: 37.8–38.6%). On the other hand, among females, the prevalence of hypertriglyceridemia continuously increased in the higher age group (*p* for trend < 0.001). Regarding the geographic region, the prevalence of hypertriglyceridemia among RTA personnel residing in the northeast was the highest, ranging from 42.6 to 45.8%, over 5 years. The prevalence of hypertriglyceridemia among RTA personnel residing in Bangkok and the north was consistent over 5 years (*p* for trend 0.340 and 0.063, respectively), while those in other regions tended to decline over half a decade (*p* for trend < 0.001).

### Demographic and behavioral factors associated with hypertriglyceridemia

Multivariable AOR from the logistic regression model is shown in Table 3. After mutually adjusting for demographic, behavioral, and cardiometabolic factors, the prevalence of hypertriglyceridemia was higher for males than that for females (AOR: 2.15; 95% CI: 2.07–2.23), RTA personnel aged 40 to 44 years compared with those aged 35 to 39 years (AOR: 1.05; 95% CI: 1.02–1.08), and those residing in the northeast (AOR: 1.15; 95% CI: 1.11–1.18) and the north (AOR: 1.05; 95% CI: 1.02–1.08) compared with those residing in Bangkok. Regarding smoking status, the prevalence of hypertriglyceridemia was higher among RTA personnel having a history of smoking, including ex-smoker (AOR: 1.16; 95% CI: 1.13–1.19), irregular current smoker (AOR: 1.48; 95% CI: 1.43–1.53), and regular current smoker (AOR: 1.66; 95% CI: 1.62–1.70), compared with those who never smoked. The prevalence of hypertriglyceridemia among RTA personnel reporting former alcohol consumption was lower when compared with abstainers, while it was higher among irregular current drinkers (AOR: 1.11; 95% CI: 1.08–1.13) and regular current drinkers (AOR: 1.32; 95% CI: 1.27–1.37). Finally, those who reported regular exercise had a lower prevalence of hypertriglyceridemia than sedentary participants (AOR: 0.87; 95% CI: 0.86–0.89).

**Table 3:** Univariable and multivariable analysis for association between demographic, behavioral and cardiometabolic factors and the prevalence of hypertriglyceridemia among RTA personnel (2017–2021)

Factors	Hypertriglyceridemia n (%)	Unadjusted Odds Ratio	95% CI	***p***-value	Adjusted Odds Ratio	95% CI	***p***-value
**Sex**							
Female	102,301 (44.3)	1			1		
Male	5295 (19.9)	3.19	3.10–3.30	< 0.001	2.15	2.07–2.23	< 0.001
**Age (years)**							
35–39	22,470 (42.2)	1			1		
40–44	22,083 (45.1)	1.12	1.10–1.15	< 0.001	1.05	1.02–1.08	0.002
45–49	18,243 (43.6)	1.06	1.03–1.09	< 0.001	0.96	0.93–0.99	0.004
50–54	21,354 (40.9)	0.95	0.92–0.97	< 0.001	0.83	0.81–0.86	< 0.001
≥ 55	23,446 (38.2)	0.85	0.83–0.87	< 0.001	0.76	0.74–0.78	< 0.001
**Regions**							
Bangkok	16,978 (37.8)	1			1		
Central	39,401 (42.0)	1.19	1.16–1.22	< 0.001	1.01	0.99–1.04	0.270
Northeast	19,863 (44.1)	1.30	1.27–1.33	< 0.001	1.15	1.11–1.18	< 0.001
North	21,620 (43.8)	1.28	1.25–1.32	< 0.001	1.05	1.02–1.08	0.003
South	9734 (39.5)	1.07	1.04–1.11	< 0.001	0.79	0.76–0.82	< 0.001
**Health scheme**							
Civil servant medical benefits	105,909 (42.0)	1			1		
Social Security	987 (26.3)	0.49	0.46–0.53	< 0.001	1.01	0.93–1.1	0.787
Universal Coverage	700 (39.8)	0.91	0.83–1.00	0.062	1.08	0.97–1.2	0.152
**Smoking status**							
Never	47,583 (36.7)	1			1		
Ex-smoker	23,133 (42.8)	1.29	1.26–1.31	< 0.001	1.16	1.13–1.19	< 0.001
Current smoker (irregular)	13,916 (49.4)	1.68	1.64–1.73	< 0.001	1.48	1.43–1.53	< 0.001
Current smoker (regular)	20,787 (50.9)	1.79	1.75–1.83	< 0.001	1.66	1.62–1.70	< 0.001
**Alcohol consumption**							
Never	20,522 (34.2)	1			1		
Ex-drinker	11,757 (37.6)	1.16	1.13–1.19	< 0.001	0.92	0.89–0.95	< 0.001
Current drinker (irregular)	62,735 (44.7)	1.55	1.52–1.58	< 0.001	1.11	1.08–1.13	< 0.001
Current drinker (regular)	10,537 (49.1)	1.86	1.8–1.920	< 0.001	1.32	1.27–1.37	< 0.001
**Regular exercise**							
No	45,354 (43.7)	1			1		
Yes	59,708 (40.4)	0.87	0.86–0.89	< 0.001	0.87	0.86–0.89	< 0.001
**Body mass index (kg/m**^**2**^**)**							
18.50–22.99	17,514 (26.6)	1			1		
< 18.50	724 (20.2)	0.70	0.64–0.76	< 0.001	0.72	0.65–0.79	< 0.001
23.00–24.99	25,659 (39.6)	1.81	1.77–1.85	< 0.001	1.66	1.62–1.70	< 0.001
25.00–29.99	48,966 (50.2)	2.79	2.73–2.85	< 0.001	2.40	2.34–2.45	< 0.001
≥ 30.00	14,733 (57.0)	3.66	3.56–3.78	< 0.001	3.00	2.90–3.11	< 0.001
**Fasting plasma glucose (mg/dL)**							
< 100	55,440 (36.0)	1			1		
100–125	28,294 (48.9)	1.70	1.67–1.73	< 0.001	1.48	1.45–1.51	< 0.001
≥ 126	14,542 (60.9)	2.76	2.69–2.84	< 0.001	2.36	2.29–2.43	< 0.001
**High blood pressure**							
No	64,786 (37.1)	1			1		
Yes	42,810 (51.4)	1.79	1.76–1.82	< 0.001	1.40	1.37–1.43	< 0.001
**Total cholesterol (mg/dL)**							
< 200	33,295 (32.5)	1			1		
≥ 200	74,301 (47.9)	1.91	1.88–1.94	< 0.001	1.98	1.94–2.02	< 0.001
**Year**							
2017	18,555 (43.4)	1			1		
2018	20,933 (42.9)	0.98	0.95–1.00	0.112	0.99	0.96–1.02	0.377
2019	23,843 (41.8)	0.94	0.91–0.96	< 0.001	0.93	0.91–0.96	< 0.001
2020	23,409 (40.3)	0.88	0.86–0.90	< 0.001	0.85	0.82–0.87	< 0.001
2021	20,856 (41.0)	0.91	0.88–0.93	< 0.001	0.85	0.82–0.87	< 0.001

### Cardiometabolic risk factors related to hypertriglyceridemia

**Fig. 3:**
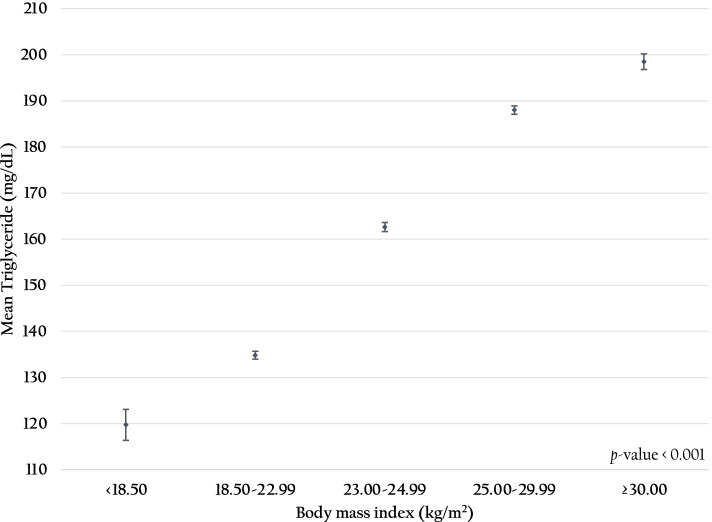
Association between body mass index and mean triglyceride among RTA personnel aged 35–60 years

**Fig. 4:**
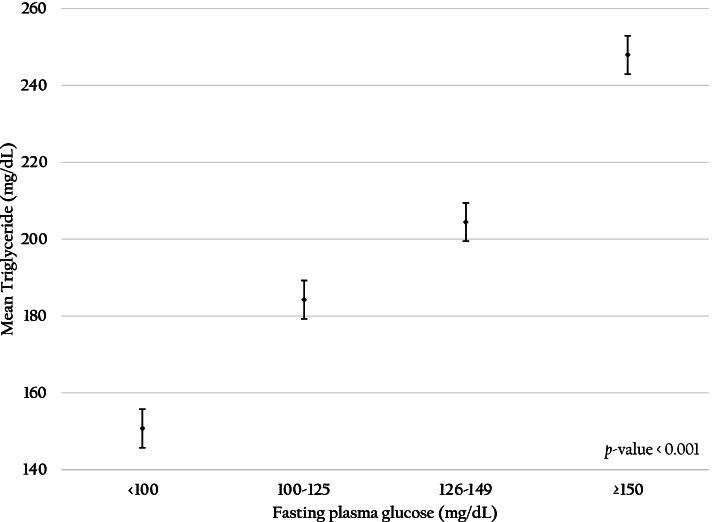
Association between fasting plasma glucose and mean triglyceride among RTA personnel aged 35–60 years

**Fig. 5:**
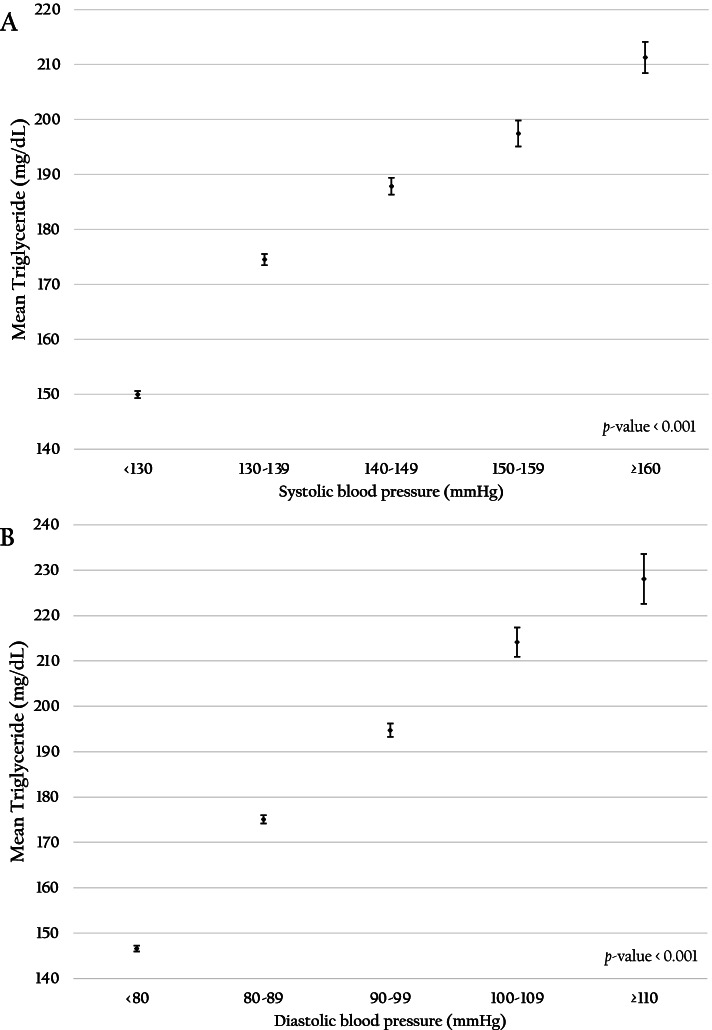
Association between systolic blood pressure (**A**), diastolic blood pressure (**B**) and mean triglyceride among RTA personnel aged 35–60 years

Figure 3 demonstrates that a higher BMI level was significantly related to rising average TG (*p-value* < 0.001). The higher the BMI recorded, the higher the prevalence of hypertriglyceridemia, especially among those with obese class I (AOR: 2.40; 95% CI: 2.34–2.45) and obese class II (AOR: 3.00; 95% CI: 2.90–3.11). Figure 4 presents the significant associations between higher FPG levels and rising average TG levels of RTA personnel (*p-value* < 0.001). Compared with the prevalence of hypertriglyceridemia among RTA personnel with FPG < 100 mg/dL, the prevalence among RTA personnel with impaired FPG (AOR 1.48; 95%CI 1.45–1.51) and hyperglycemia were higher (AOR 2.36; 95%CI 2.29–2.43). Regarding BP, hypertriglyceridemia was significantly higher among RTA personnel with SBP ≥ 140 mmHg or DBP ≥ 90 mmHg than those with BP < 140/90 mmHg (AOR 1.40; 95% CI 1.37–1.43). Figure 5 illustrates that rising SBP (5A) and DBP (5B) were significantly related to higher average TG levels of RTA personnel (*p-value* < 0.001). Finally, compared with the prevalence of hypertriglyceridemia among RTA personnel with TC < 200 mg/dL, the prevalence among RTA personnel with hypercholesterolemia was higher (AOR 1.98; 95%CI 1.94–2.02).

## Discussion

From 2017 to 2021, we successfully enrolled 257,683 RTA personnel between the ages of 35 and 60 years nationwide. This study represented the most extensive epidemiologic study of hypertriglyceridemia prevalence among RTA personnel in Thailand for over half a decade. Our finding demonstrates the high prevalence of hypertriglyceridemia and identifies associated behavioral risk factors, including smoking, current alcohol consumption, and sedentary behavior. Furthermore, we explored the association between hypertriglyceridemia and related cardiometabolic risk factors, including higher BMI, higher FPG, high BP, and high TC in this study population.

According to the NHES VI in 2019, the prevalence of hypertriglyceridemia in Thai adults aged ≥ 15 years was 36.0% [11]. Compared with the NHES VI, the overall hypertriglyceridemia prevalence among RTA personnel was higher (41.8%); however, the present study included participants aged at least 35 years. Furthermore, the National Health Survey in China [6], the US [7] and Russia [8] demonstrated a lower prevalence of hypertriglyceridemia among adults aged 20 years accounting for 13.1, 25.9, and 29.2%, respectively. On the other hand, we found that the prevalence of hypertriglyceridemia slightly decreased from 43.4% in 2017 to 40.3% in 2020 and then rose to 41.0% in 2021. Conversely, the NHES V in 2014 [10] and VI in 2019 [11] indicated that hypertriglyceridemia among Thai adults rose by 5% over 5 years. The present study found that the prevalence of hypertriglyceridemia among males in this study was comparable with that among Thai males in the NHES VI; however, among females, it was relatively low (19.9%) when compared with that in the NHES VI (30.7%) [11]. Our finding reported that the prevalence of hypertriglyceridemia among males was significantly higher than that among females, which was compatible with the recent evidence from the NHES VI [11] and data from the US [18], Russia [8] and Pacific Islands [19]. However, another study among hill tribes in Thailand documented that sex was not associated with hypertriglyceridemia [20].

We observed that the age of participants was associated with the prevalence of hypertriglyceridemia. Compared with male participants aged 35 to 39 years, hypertriglyceridemia among males aged 40 to 44 years was relatively high. However, the prevalence of hypertriglyceridemia among males decreased with higher age groups, especially those aged 55 years. Conversely, the prevalence of hypertriglyceridemia among females was continuously incremental with higher age groups. This pattern was similar to that of the NHES VI [11] and the population-based study in Russia [8].

We also discovered that RTA personnel residing in the northeast showed the highest prevalence of hypertriglyceridemia compared with those residing in other regions, which agreed with the findings from the NHES VI [11]. It may be explained by the cultural context of the Thai population residing in the northeast and the north consuming glutinous rice, a staple food item in this region of the country. Compared with nonglutinous rice, the staple food of the other parts of Thailand, glutinous rice has a high glycemic index and high carbohydrate content contributing to hypertriglyceridemia [20, 21]. These findings suggested that hypertriglyceridemia in RTA personnel, particularly males aged 40 to 44 years and those residing in the northeast and north, should be identified and treated to reduce the risk of coronary heart disease and ischemic stroke [5, 22–24].

Cigarette smoking is a known behavioral risk factor for hypertriglyceridemia [25–27]. We found that the prevalence of hypertriglyceridemia among current smokers, especially among regular current smokers, was higher when compared with those who never smoked. This phenomenon may be attributed to increased oxidative stress by the components of cigarettes, and, consequently, increased levels of triglycerides [28–31]. Furthermore, our findings indicated that the prevalence of hypertriglyceridemia among former smokers was relatively high, which agreed with a related study in Korea [26]. Nevertheless, another study in Iran reported a reverse association between former smoking and hypertriglyceridemia (OR: 0.62; 95% CI: 0.46–0.84) [32].

Our study found that the prevalence of hypertriglyceridemia among study participants who currently consume alcohol was higher than that among abstainers. Several population studies in Switzerland, Ireland, and Korea confirmed that alcohol consumption was associated with rising plasma TG [33–35]. On the other hand, some studies indicated a J-shaped association between alcohol consumption and plasma TG [36–38]. A related study documented that consumption of 3 to 20 alcoholic drinks weekly was associated with lower plasma TG than consumption of more than 20 alcoholic drinks/weekly or abstainers [38]. The NHES VI reported that current alcohol consumption among Thai adults was 44.6, 59.0, and 31.0% in total, males, and females, respectively [11]. Compared with the NHES VI, our finding indicated that the prevalence of current alcohol consumption among RTA personnel (approximately 64%) was high; thus, current alcohol consumption may play an essential role in contributing to hypertriglyceridemia in this population. Furthermore, we observed that the study participants who were former drinkers were less likely to have hypertriglyceridemia. Our study suggested that alcohol consumption and cigarette smoking were potential behavioral risk factors related to hypertriglyceridemia. Thus, reducing or stopping cigarette smoking and alcohol consumption should be encouraged to attenuate the prevalence of hypertriglyceridemia and alleviate the risk for ASCVD [39–41].

RTA personnel may be more physically active than the general civilian population. Nevertheless, the RTA has several departments with various characteristics of duty; thus, various levels of physical activities exist across our study population [11]. Our study demonstrated that the prevalence of hypertriglyceridemia among study participants who reported regular exercise was lower than that of those reporting sedentary behavior. In general, the effects of aerobic exercise were observed on reduced TG (− 12 mg/dL, 95% CI: − 16 to − 8 mg/dL among males; − 4 mg/dL, 95% CI: − 8 to 0 among females) [42]. In addition, a related randomized controlled trial indicated a beneficial effect of exercise on attenuating TG level, clearly with a high amount of moderate-to-intense exercise [43]. Therefore, our study suggested that regular exercise and progressive resistance training may be feasible to alleviate TG levels in this population [44]. However, vigorous exercise can rarely cause heat-related illness and acute coronary syndrome [45, 46], so physical exercise should be performed appropriately based on relevant evidence [47].

Multiple evidence indicated that cardiometabolic risk factors, including obesity, high FPG, high BP, and high TC, affected several cardiovascular diseases, i.e., stroke, ischemic heart disease, and atrial fibrillation [39, 48–52]. Our findings revealed a linear relationship between hypertriglyceridemia and higher BMI. Additionally, we observed that the average TG level of study participants presenting overweight was approximately 160 mg/dL. Furthermore, the average TG level among individuals with BMI ≥ 30 kg/m^2^, which is obese class II according to the Asia-Pacific perspective, was just below 200 mg/dL. This phenomenon could be described by abnormal triglyceride metabolism, including increased hepatic very-low-density lipoprotein production and decreased triglyceride hydrolysis, which was affected by adipocyte hypertrophy among individuals with higher BMI [53, 54]. Furthermore, the evidence documented that hypertriglyceridemia is also a hallmark of dyslipidemia in obesity [54]. Therefore, our study suggested that weight management through lifestyle change, including physical exercise and maintaining a healthy diet, should be encouraged in this population [55, 56].

We found that the prevalence of hypertriglyceridemia among the study participants with impaired FPG and hyperglycemia was significantly higher than those with FPG less than 100 mg/dL; additionally, the average TG level among individuals with FPG 150 mg/dL was elevated to approximately 250 mg/dL. A ten-year retrospective cohort study in Thailand indicated that hypertriglyceridemia was significantly associated with the incidence of type 2 diabetes (T2D) [57]. Alternatively, hyperglycemia or T2D can lead to hypertriglyceridemia via several mechanisms, including increased flux free fatty acid to the liver and decreased activation of lipases [58]. However, either hypertriglyceridemia or hyperglycemia alone suggests that they have an independent effect on the endothelial cells, contributing to the atherosclerotic process [59]. Therefore, our study indicated that primary prevention of T2D and its sequel should be promoted, especially in one-third of study participants with FPG ≥ 100 mg/dL.

We observed that RTA personnel with high BP (SBP ≥ 140 mmHg or DBP ≥ 90 mmHg) tended to have hypertriglyceridemia 1.4 times when compared with those with normal BP, which was compatible with the related studies in the US [60] and Taiwan [61]. Furthermore, the present study also demonstrated a positive association between mean TG and BP as a linear relationship. A recent animal model study indicated that hypertriglyceridemia may have enhanced resistance arterial responsiveness and increased BP [62]. The robust evidence documented that both hypertriglyceridemia and hypertension can play an independent role in increasing the risk for ASCVD [3, 39, 63]. Therefore, our results suggested that hypertriglyceridemia and high BP in this population, especially those with combined hypertriglyceridemia and high BP, should be recognized and effectively managed to prevent further complications such as stroke, ischemic heart disease, and premature death.

A significant relationship between hypercholesterolemia and hypertriglyceridemia was observed. This finding, consistent with the outcome of a related population-based study in Spain, indicated that hypercholesterolemia is the independent factor associated with hypertriglyceridemia (AOR 4.6) [64]. Evidence documented that both hypercholesterolemia and hypertriglyceridemia are independent risks for ASCVD [3, 5, 65, 66]. Furthermore, in the present study, approximately one-third of participants had a combination of hypercholesterolemia and hypertriglyceridemia. Thus, our findings suggested that this issue should be considered, and effective management such as lipid-lowering therapy may help reduce the risk for ASCVD [67]. However, nonpharmacological methods such as lifestyle modification should be initiated, as well as long-term measures to address this issue [68].

Some limitations were encountered in this study. Firstly, the study employed a serial cross-sectional design; thus, the results could present only the association between hypertriglyceridemia and related factors. Second, the present study was conducted among RTA personnel comprising a higher proportion of male participants (approximately 90%); however, the results reported a real-world situation in this study population. Third, due to the observational study using previously collected data from a health examination database, data on some variables were missing, including fasting plasma glucose (8.6%), exercise (2.4%), smoking status (1.9%), and alcohol consumption (1.7%). Although we were aware of missing data, the present study consisted of a large sample size to include the existing data in the analysis. Fourth, some variables were collected very broadly, e.g., the total number of cigarettes smoked and smoking frequency. In addition, the present study did not contain the intensity and frequency of alcohol use; thus, we cannot explore this association between the intensity of alcohol consumption and hypertriglyceridemia. Similarly, we did not have detailed data on the intensity, or type of exercise. Nonetheless, the available data provided valuable evidence regarding the associations between these behavioral factors and the prevalence of hypertriglyceridemia. Finally, regarding the existing literature that abdominal obesity contributes to an increased ASCVD [69, 70], unfortunately, waist circumference and abdominal obesity were not included in the analysis. Therefore, we do not have an opportunity to explore the relationship between abdominal obesity and hypertriglyceridemia.

Our study also encompassed considerable strengths, including representing a large sample of RTA personnel. Thus, our data provided valuable insights into the prevalence of hypertriglyceridemia and its related behavioral and cardiometabolic risk factors in Thailand. Furthermore, these data may produce strategies for the primary prevention of ASCVD and premature death in this population.

## Conclusion

Our data demonstrated that hypertriglyceridemia is a frequent health issue, especially among males, participants aged 40 to 44 years, and RTA personnel residing in the northeast and the north. Alcohol consumption, cigarette smoking, and sedentary behavior played an essential role in the prevalence of hypertriglyceridemia in this population. In addition, cardiometabolic risk factors, including higher BMI, high FPG, high BP, and high TC, were significantly related to hypertriglyceridemia. Both behavioral and cardiometabolic risk factors are potential targets for intervention to enhance the primary prevention of sequelae of hypertriglyceridemia, including ischemic heart disease and stroke.

## Data Availability

Data cannot be shared publicly because the data set contains identifying information; additionally, the data belong to the Royal Thai Army Medical Department. Thus, ethics restrictions exist concerning the data set. Data are available from the Royal Thai Army Medical Department, Bangkok, Thailand for researchers meeting the criteria to access confidential data.

## Abbreviations

TG: Triglyceride
HT: Hypertension
NCDs: Noncommunicable diseases
T2D: Type 2 diabetes
ASCVD: Atherosclerotic cardiovascular diseases
BMI: Body mass index
FPG: Fasting plasma glucose
BP: Blood pressure
RTA: The Royal Thai Army
RTAMED: The Royal Thai Army Medical Department
NHES: National Health Examination Survey
AOR: Adjusted odds ratio
CI: Confidence interval
SD: Standard deviation
